# Visits to Pediatric Clinics by Adult Patients: A Nationwide Survey in Taiwan

**DOI:** 10.3390/ijerph15071538

**Published:** 2018-07-20

**Authors:** Kang-Lung Lee, An-Min Lynn, Tzeng-Ji Chen, Ling-Yu Yang, Shu-Chiung Chiang

**Affiliations:** 1 Department of Radiology, Taipei Veterans General Hospital, No. 201, Sec. 2, Shi-Pai Road, Taipei 112, Taiwan; miguelkllee@gmail.com; 2 School of Medicine, National Yang-Ming University, No. 155, Sec. 2, Linong Street, Taipei 112, Taiwan; tjchen@vghtpe.gov.tw; 3 Master of Public Health Degree Program, College of Public Health, National Taiwan University, No. 17, Xuzhou Road, Taipei 100, Taiwan; nashsaka@hotmail.com; 4 Department of Family Medicine, Chang Gung Memorial Hospital, No. 5, Fuxing Street., Guishan District, Taoyuan City 33305, Taiwan; 5 Department of Family Medicine, Taipei Veterans General Hospital, No. 201, Sec. 2, Shi-Pai Road, Taipei 112, Taiwan; 6 Institute of Hospital and Health Care Administration, National Yang-Ming University, No. 155, Sec. 2, Linong Street, Taipei 112, Taiwan; scchiang0g@gmail.com; 7 Department of Medical Education, Taipei Veterans General Hospital, No. 201, Sec. 2, Shi-Pai Road, Taipei 112, Taiwan; 8 Department of Pediatrics, Taipei Veterans General Hospital, No. 201, Sec. 2, Shi-Pai Road, Taipei 112, Taiwan; 9 Department of Financial Engineering and Actuarial Mathematics, Soochow University, Taipei 100, Taiwan

**Keywords:** pediatrics, national health insurance, ambulatory visits, adults

## Abstract

Pediatricians are trained to provide non-surgical medical care to children. Improvements in medical treatments and surgical techniques have extended the survival of children with congenital diseases and chronic illnesses. Consequently, pediatricians may provide continuous medical service to their patients into adulthood. Meanwhile, as Taiwan’s birth rate has fallen to one of the lowest in the world, pediatricians are encountering growing competition. As a source of continued revenue, pediatricians could also provide medical care to adults with common diseases and patients with adult-onset chronic diseases. The aim of this study was to investigate the pattern of adult ambulatory visits to pediatric clinics recorded by Taiwan’s National Health Insurance (NHI) system during 2000 to 2011. From 1/500 sampling datasets, we found that adult ambulatory visits to pediatric clinics rose steadily and statistically significantly from 16% of total visits to pediatric clinics in 2000 to 32% in 2011. Analysis of the diagnoses associated with adult ambulatory visits to pediatric clinics indicated that the most common diagnoses for such patients at academic medical centers were chronic illnesses, including epilepsy, cardiac and circulatory congenital anomalies, and diabetes. Meanwhile, at physician clinics, airway infections/diseases and gastroenteritis were the most common diagnoses. In an era of low birth rates, our findings contribute to an evidence-based discussion and provide new information that may assist in healthcare policymaking.

## 1. Introduction

Traditionally, pediatricians have been trained to provide non-surgical medical care to children younger than 18 years old [1]. However, owing to the improvements in medical treatments and surgical techniques, children with chronic illness are surviving longer [2,3,4,5]. As they grow up, these patients often encounter difficulty transitioning from pediatric to adult medical care [6,7,8]. Some choose to stay in the pediatric medical care system into adulthood because they have built good long-term relationships with their doctors, leading to pediatricians treating adult patients with chronic illness.

Because the birth rate in Taiwan is among the lowest in the world, pediatricians in Taiwan have experienced greater competition with other specialists in the last decade [9], which has provided an incentive for them to provide care to adults. Although the role that pediatricians play in the ambulatory care of children has been examined [1,10], there is little information about why adults receive care at pediatric clinics. The aim of this study was to investigate the pattern of adult ambulatory visits to pediatric clinics based on the system records of Taiwan’s National Health Insurance (NHI) from 2000 to 2011. We analyzed the ages of the patients and the diagnoses associated with each visit. This work is relevant for planning the distribution of the physician workforce and may provide information to support healthcare policymaking. In an era of low birth rates, our results provide empirical data that can inform the role pediatricians should play in ambulatory care.

## 2. Materials and Methods

### 2.1. Data Collection

This study was approved by the Institutional Review Board of Taipei Veterans General Hospital, Taipei, Taiwan (VGHIRB No.: 2013-04-005E). The NHI program was established in Taiwan in 1995 and had a coverage rate >99.5% of the whole population in Taiwan (23,074,487 beneficiaries) by the end of 2011. The National Health Insurance Research Database (NHIRD, http://nhird.nhri.org.tw/) contains the NHI health claim data, managed by the National Health Research Institutes, and has been used in a variety research fields [11].

### 2.2. Study Population

For our analysis, we obtained the NHIRD systematic sampling CD dataset, which is comprised of 0.2% of the ambulatory care expenditures, by visit (CD); this dataset was extracted by monthly systematic sampling from 2000 to 2011 [12]. The ambulatory care records include encrypted personal identification numbers, dates of birth, gender, dates of visits, and up to three diagnoses coded according to the International Classification of Disease, Ninth Revision, Clinical Modification (ICD-9-CM) [13].

From the medical records samples, we extracted and analyzed 141,354 adult ambulatory visits to pediatric clinics. Visit years were categorized into three periods: 2000–2003, 2004–2007, and 2008–2011. The patients were classified into three age groups (in years): 18–39, 40–64, and ≥65. Visits were classified according to four hospital levels: academic medical center, metropolitan hospital, local community hospital, and physician clinic. Clinical Classification Software, 2010 version, was used to classify the diagnoses into clinically meaningful categories [14].

### 2.3. Statistical Analysis

Perl software, version 5.20.2 (Perl Foundation, Holland, MI, USA), was used to retrieve data from the NHIRD. The data were analyzed in Microsoft Office Excel 2013 (Microsoft Corporation, Washington, DC, USA) with respect to frequency of ambulatory pediatric clinic visits in relation to the aforementioned patient age groups and hospital levels. The top ten most common adult ambulatory care diagnoses were categorized by hospital level within each of the three aforementioned time periods. R 3.4.2 (R Foundation for Statistical Computing, Vienna, Austria), was used for conducting statistical models. The Poisson regression was employed to evaluate for the incidence rate ratio and the corresponding interval estimates (95% confidence intervals, CIs), with the overall top ten most common diagnoses of adult ambulatory visits to pediatric clinics and year of diagnosis (categorized as 2000–2003, 2004–2007, 2008–2011) as response variables and the natural logarithm number of adult ambulatory visits to all specialists as the offset term. A *p*-value of less than 0.05 was employed to determine statistical significance.

## 3. Results

A total of 596,316 ambulatory visits to pediatric clinics from 2000 to 2011 were retrieved from the sampling data. Of these visits, 454,782 (76.3%) involved pediatric patients (age < 18 years) and 141,354 (23.7%) involved adult patients (age ≥ 18 years). The latter group of adult patient visits were included in this study. Stratifying the data by age group revealed that patients aged 18–39 years accounted for the highest proportion (52.2%, *n* = 73,900) of adult ambulatory visits to pediatric clinics, followed by patients aged 40–64 years (36.2%, *n* = 51,172); patients aged 65 years and older accounted for the lowest proportion (11.5%, *n* = 16,282).

**Figure 1:**
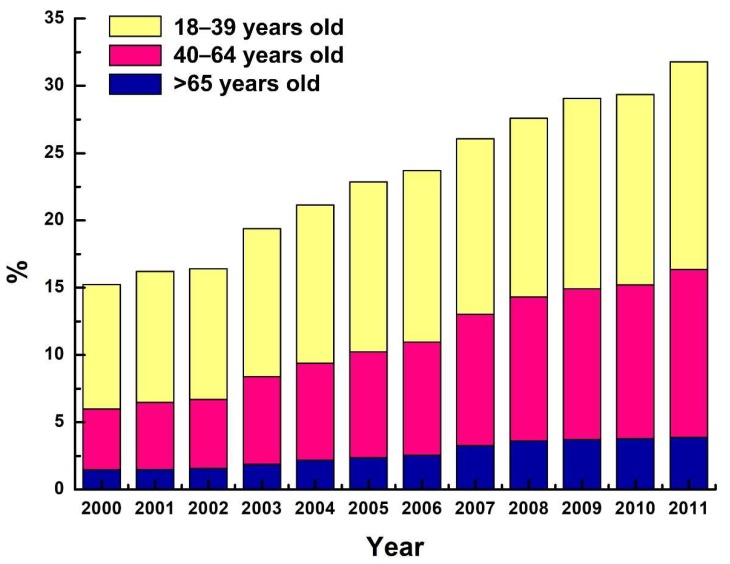
Annual proportions of adult ambulatory visits to pediatricians by patient age, 2000 to 2011.

The total number of ambulatory visits to pediatric clinics, regardless of age, has increased year by year. However, further analysis revealed that the proportion of children’s ambulatory visits to pediatric clinics decreased steadily year to year (from 84.8% in 2000 to 68.2% in 2011). On the contrary, the proportion of adult ambulatory visits to pediatric clinics, regardless of age, increased steadily year by year (Figure 1). From the 2000–2003 period to the 2008–2011 period, there were 1.7-fold, 2.8-fold, and 2.7-fold increases in the proportions of adult patients utilizing pediatric ambulatory services in the 18–39, 40–64, and ≥65-year-old age groups, respectively (Table 1).

In this study sample, physician clinics were the major ambulatory care providers for adult patients, accommodating 96.8% (*n* = 136,791) of adult ambulatory visits to pediatric clinics, followed by academic medical centers (1.3%), local community hospitals (1.0%), and metropolitan hospitals (0.9%). We assembled a top 10 list for the most common diagnoses of adult ambulatory care based on an analysis of the first diagnosis code in each medical record and categorized the data by hospital level. The most common diagnosis categories overall were acute inflammation and infection-related diseases. The top three most common diagnoses did not change across the three analyzed time periods. The top three diagnoses across the three periods were upper respiratory infection (45.5%), followed by acute bronchitis (9.3%) and acute or chronic tonsillitis (5.5%).

Differences among the most common diagnoses over time emerged when hospital level categories were considered (Table 2, Table 3 and Table 4). Ranking figures of top ten most common diagnoses of adult ambulatory care were assembled (Supplementary Materials Figures S1–S4). For academic medical centers, epilepsy remained the most common diagnosis over the full study period. Although their relative rankings changed slightly over time, the following three diagnoses remained in the range of second to fifth most common from 2000 to 2011: congenital cardiac and circulatory anomalies; deficiency and other anemia; and jaundice. It is noteworthy that upper respiratory disease, which was the second most common diagnosis (*n* = 45) during the period of 2000 to 2003 and the third most common diagnosis (*n* = 38) during the period of 2004 to 2007, became the tenth most common diagnosis (*n* = 22) during the period of 2008 to 2011. Meanwhile, the frequency of diabetes diagnoses increased over the 12-year study period, rising from the 28th most common diagnosis (*n* = 1) in the period of 2000 to 2003, to the tenth most common diagnosis (*n* = 19) in the period of 2004 to 2007, and to the fifth most common diagnosis (*n* = 33) in the period of 2008 to 2011. Systemic lupus erythematosus remained the sixth (*n* = 14, 2000–2003 and *n* = 30, 2008–2011) or seventh (*n* = 27, 2004–2007) most common diagnosis in the study period (Table 2, Table 3 and Table 4) (Figures S1–S4).

At metropolitan hospitals and local community hospitals, the most common diagnoses during the study period were related mainly to inflammation, infection, and allergic diseases, especially airway infections. Although epilepsy was the sixth (*n* = 11) and seventh (*n* = 15) most common diagnosis at metropolitan hospitals in the periods of 2000 to 2003 and 2004 to 2007, respectively, it was no longer within the top 10 diagnoses in the period of 2008 to 2011. Conversely, jaundice was not a top ten diagnosis in 2000 to 2003, but in 2004 to 2012 it became the most common diagnosis at metropolitan hospitals and the second most common diagnosis at local community hospitals (Table 2, Table 3 and Table 4) (Figures S1–S4).

Overall, airway infections/diseases and gastroenteritis were consistently common diagnoses at adult ambulatory visits to pediatrician clinics during the study period in this survey. Upper airway infections were the most common diagnosis during the study period of 2000 to 2011, followed by acute bronchitis and acute/chronic tonsillitis. Noninfectious gastroenteritis was the fourth (2000–2003) or fifth (2004–2011) most common diagnosis. It is noteworthy that the incidence of essential hypertension among adults seeing pediatricians rised. Essential hypertension represented the tenth most common diagnosis, accounting for 0.9% of ambulatory service care visits during the period of 2000–2003. Then, its proportion rose steadily, reaching 1.6% in the period of 2004–2007 and 2.9% in the period of 2008–2011. Essential hypertension was ranked the eighth most common diagnosis at physician clinics during the period of 2008–2011 (Table 2, Table 3 and Table 4).

**Figure 2:**
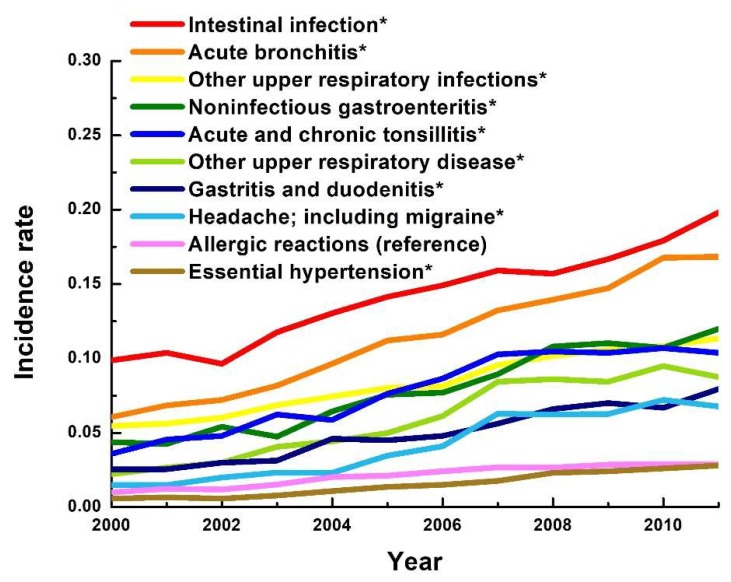
Trends of incidence rate of the overall top ten most common diagnoses of adult ambulatory visits to pediatric clinics. (ref = reference level; * *p* < 0.05).

The incidence rate of ambulatory visits to pediatric clinics significantly increased from 2000 to 2011. The incidence rates of the top ten most common diagnoses all show steady increase from 2000 to 2011. When allergic reaction was set as reference level, all the top ten most common diagnoses, except essential hypertension, had significantly higher incidence rates than allergic reaction did. Special attention should be paid to essential hypertension, as the incidence rate ratio of essential hypertension over allergic reaction was 0.50 in 2000, and the ratio steadily increased to 0.97 in 2011 (Figure 2 and Table S1).

## 4. Discussion

In our study, we found that the proportion of adults among ambulatory patients visiting pediatric clinics rose steadily with statistically significantly differences observed over time. This increase might reflect the falling birth rate in Taiwan. Depopulation is a global trend and garnering substantial public attention in East Asia [9]. In 2010, the total birth rate in Taiwan fell to 0.89, the lowest birth rate in the world [9]; since then it has remained among the lowest three. This decreasing birth rate trend has not been reversed despite policy efforts by the Taiwanese government to encourage childbearing, including a childbirth subsidy, maternity pension, children’s education subsidy, and children’s healthcare subsidy [15,16,17]. Due to depopulation, pediatricians in Taiwan are facing unprecedented levels of competition with other physicians. To compensate, pediatricians can elongate their care into adulthood, especially for long-term patients with chronic diseases they have been treating since childhood.

This practice is well justified by challenges that some young adult chronic disease patients face in the transition from pediatric to adult medical care, including the occurrence of undesirable health outcomes, such as a worsening of glycemic control for teenaged patients with diabetes [18,19]. Deterioration of health status has also occurred for teens diagnosed with pediatric epilepsy and teens who received cardiovascular surgery or other interventions in childhood [20,21,22,23]. Whereas some countries have a barrier between pediatric and adult care, which is managed by bureaucrats [24], in the Taiwan NHI program, people are free to choose their physicians for ambulatory visits without strict regulations of referral requirements [1]. In other words, as the population of children shrinks, pediatricians have some incentive to expand into adolescent and adult medical care to ensure the long-term quality of care that their patients receive and to maintain a full medical practice. Pediatricians might also explore a transboundary market, such as treating some common adult-onset diseases.

We found that the most common diagnoses made by pediatricians differed among hospital levels. At academic medical centers, chronic illnesses—such as epilepsy, congenital heart disease, anemia, jaundice, systemic lupus erythematosus, and diabetes—were the most common diagnoses for adult ambulatory visits to pediatric clinics. On the one hand, as the population is aging, pediatricians staffing in hospitals would have some incentive to provide medical services to young adults and/or patients with chronic conditions who are accustomed to the pediatric medical delivery system since childhood. On the other hand, improvements in medical treatments and surgical techniques have enabled patients with congenital diseases to survive longer [20,21,22,23]. For example, most congenital heart disease mortality has been delayed into adulthood [2,24,25,26]. Nevertheless, many children reach adolescence with little understanding of the implications of their cardiac condition [27]. Patients who have grown up with congenital heart disease have particular medical service needs when transitioning from pediatric to adult care [26]. Many guidelines and training programs for grown-up congenital heart disease have been set up, and pediatric cardiologists always play very important roles in that field [2,25,27].

The principle of medical specialties indicates that pediatric care should transition to adult care when patients reach 18 years old [1,19]. However, for patients with epilepsy, transferring from pediatric to adult care has shortcomings and adult neurologists often have insufficient information about new patients’ past and current medical conditions [3]. Additionally, these patients may prefer to delay the need to develop new patient-physician relationships and move from a familiar to an unknown ward culture [6]. Staying in the pediatric medical delivery system can enable them to avoid these transition difficulties.

With the increasing prevalence of type 1 and type 2 diabetes mellitus in children worldwide [4,5,28], the demand for medical care for diabetic children is increasing. As these diabetic children grow up, they are expected to transition from pediatric to adult medical care [7,8]. Nonetheless, similar to the difficulties faced by epileptic patients, diabetic patients may prefer to continue being treated by their pediatricians. Such a preference could explain, at least in part, why diabetes rose from a relatively uncommon diagnosis (28th) to the fifth most common diagnosis for adult ambulatory visits to pediatric clinics in the period of time analyzed in this study. In summary, for those patients with congenital diseases or chronic diseases diagnosed in their childhood or infancy, these patients prefer to stay in the pediatric medical delivery system for continuity. On the supply side, faced with a declining pediatric population, pediatricians have incentive to provide medical services to these patients. As the effect of the demand side and supply side combined, the proportion of adult ambulatory visits to pediatricians staffing in hospitals has steadily increased.

Although 74.3% of children’s ambulatory visits to pediatricians occurred at physician clinics, notably, 96.8% of adult ambulatory visits to pediatricians occurred at physician clinics, a rate vastly higher than at the other three site types [1], and also much higher than the proportion of ambulatory visits to physician clinics with other types of specialists [11,29]. Indeed, it is reasonable to suppose that the 12.4% growth in the number of physician clinics in Taiwan from 2004 to 2011 may have played a role in motivating pediatricians to expand their medical service offerings to adults due to intensifying competition.

Although chronic diseases occupy a large proportion of medical services delivered at adult ambulatory visits to pediatricians in academic medical centers, most adult patients appeared to seek medical care for airway infections/diseases and gastroenteritis in physician clinics. Similar observations were reported in a previous study in which upper respiratory tract infection was found to be the most common diagnosis made by pediatricians at children’s ambulatory visits [1,10]. In our study, we found that the diagnosis of essential hypertension increased steadily over the examined years. This increase might be explained in part by population aging, which raises the prevalence of chronic diseases, such as hypertension, and associated healthcare needs [30].

In a society with an aging population and a decreasing birth rate, we postulated that pediatricians may provide more medical services to adults. The present results are consistent with that supposition. Although pediatricians may have the opportunity to care for some adult patients with chronic illnesses diagnosed in childhood during their residencies, the current residency training program for pediatricians does not include an adult ward/ambulatory rotation or the distinct opportunity to evaluate and treat sick adults, especially geriatric patients. Nevertheless, the addition of adult or geriatric medical training per se to a pediatric residency would be on its face irrational. Therefore, adding formal adult and geriatric medicine training before doctors-in-training begin a pediatric residency, such as in a postgraduate year, may improve adult ambulatory care quality. The effects of the declining birth rate on the population are approaching irreversibility. Therefore, policy makers should re-consider what role pediatricians could and should play in the healthcare arena after completing their residency training. Although a theoretical system dynamics model for the Taiwanese pediatric workforce has been reported [31], it has not yet been determined empirically how many pediatricians should be trained. This question merits further discussion and study.

This study had some limitations. First, we did not obtain data on quality of care or cost effectiveness, such as adequacy of medication for different diagnoses, medication dosage, or medical costs per visit. Therefore, we could not evaluate the quality of care provided by pediatricians to adult patients. Second, by analyzing a sampling of claim data, we could not know the exact final diagnosis associated with every ambulatory visit. Third, there is sometimes a time gap between births and formal birth registrations in Taiwan. In this time gap, neonates might utilize medical services via their parents’ NHI identity in so-called dependent visits. Therefore, some of the NHI claims data-associated neonatal diagnoses may be linked with parents’ NHI identities. This phenomenon could potentially have introduced some bias into our analyzed datasets. In our clinical experience in Taiwan, this phenomenon is most prevalent in inpatient departments shortly after neonates are born. Generally, families register a newborn child’s birth very soon after the neonate has been discharged. It is illegal to delay birth registration and doing so would prevent the child from accessing government funded social welfare benefits. Consequently, dependent visits for newborns brought to pediatric clinics are not typical. However, because we used the systematic sampling CD dataset, we cannot trace all ambulatory and inpatient claim data for each patient. Hence, we could not clarify this bias absolutely. Nonetheless, based on the aforementioned circumstances, we believe this possible caveat should not affect our conclusions.

## 5. Conclusions

The provision of adult ambulatory medical care by pediatricians increased steadily from 2000 to 2011 in Taiwan, accounting for about one-third of ambulatory visits to pediatric clinics in 2011. At academic medical centers, chronic illnesses were the most common diagnoses made by pediatricians for adult ambulatory visits, whereas airway infections/diseases and gastroenteritis were the most common diagnoses at physician clinics. We observed increasing trends for diabetes and essential hypertension diagnoses by pediatricians in academic centers and physician clinics, respectively. In the modern low birth rate era, there is a need to re-examine the role that pediatricians should play in adult ambulatory care.

## Figures and Tables

**Table 1:** Number of ambulatory visits to pediatric clinics by patient age from the 1/500 sampling datasets, 2000 to 2011.

Age Group, Years	Visit Year											
Hospital Level	2000	2001	2002	2003	2004	2005	2006	2007	2008	2009	2010	2011
0–17												
Academic medical center	4296	4092	4131	2928	3021	2832	2456	2517	2387	2331	2449	2250
Metropolitan hospital	4983	5065	5607	4440	4532	3924	3433	3418	3508	3628	3448	3653
Local community hospital	3298	3186	3278	2703	2870	2508	2205	2250	2167	2214	2252	2293
Physician clinic	26,090	24,596	25,113	25,269	27,443	29,231	26,895	30,432	29,477	30,315	31,200	32,168
18–39												
Academic medical center	94	98	105	104	115	122	117	137	136	139	167	198
Metropolitan hospital	37	39	70	63	118	115	91	102	84	113	113	100
Local community hospital	40	51	57	56	89	98	95	86	101	96	81	138
Physician clinic	4039	4100	4202	4605	5314	5961	5530	6482	6576	7327	7512	8687
40–64												
Academic medical center	18	20	18	15	26	14	23	17	12	15	24	25
Metropolitan hospital	8	12	17	19	22	17	13	16	24	23	15	26
Local community hospital	17	21	17	30	29	23	25	25	30	29	27	37
Physician clinic	2022	2146	2285	2793	3389	3870	3795	5041	5477	6014	6306	7285
>65												
Academic medical center	6	4	8	6	6	1	4	6	2	7	5	3
Metropolitan hospital	2	2	3	7	4	3	4	6	2	3	7	2
Local community hospital	10	11	9	8	12	7	9	11	11	22	15	21
Physician clinic	650	640	699	801	1028	1174	1157	1689	1861	1985	2076	2273
Total	45,610	44,083	45,619	43,847	48,018	49,900	45,852	52,235	51,855	54,261	55,697	59,159

**Table 2:** Top 10 most common adult ambulatory care diagnoses by hospital level, 2000–2003.

Rank	Academic Medical Center	Metropolitan Hospital	Local Community Hospital	Physician Clinic
1	Epilepsy	URD	URI	URI
2	URD	URI	URD	Acute bronchitis
3	CVCA	Acute bronchitis	Acute bronchitis	Tonsillitis
4	Anemia	Tonsillitis	Asthma	Gastroenteritis
5	URI	Pneumonia	Allergic reactions	Influenza
6	Asthma	Epilepsy	Pneumonia	Intestinal infection
7	SLE	Asthma	Tonsillitis	Gastritis and duodenitis
8	Endocrine	Allergic reactions	Gastroenteritis	Allergic reactions
9	Acute bronchitis	Gastroenteritis	Hypertension	URD
10	Pneumonia	Anemia	CVD	Hypertension

Epilepsy = epilepsy, convulsions; URD = other upper respiratory disease; CVCA = cardiac and circulatory congenital anomalies; Anemia = deficiency and other anemia; URI = other upper respiratory infections; SLE = systemic lupus erythematosus and connective tissue disorders; Endocrine = other endocrine disorders; Pneumonia = pneumonia (except that caused by tuberculosis or sexually transmitted disease); Tonsillitis = acute and chronic tonsillitis; Gastroenteritis = noninfectious gastroenteritis; Hypertension = essential hypertension; CVD = acute cerebrovascular disease.

**Table 3:** Top 10 most common diagnoses of adult ambulatory care by hospital level, 2004–2007.

Top 10 Order	Academic Medical Center	Metropolitan Hospital	Local Community Hospital	Physician Clinic
1	Epilepsy	Jaundice	URI	URI
2	CVCA	URI	Jaundice	Acute bronchitis
3	URD	URD	Acute bronchitis	Tonsillitis
4	Anemia	Acute bronchitis	Tonsillitis	Gastroenteritis
5	Jaundice	Allergic reactions	URD	URD
6	SLE	Asthma	Admission	Gastritis and duodenitis
7	Asthma	Epilepsy	Allergic reactions	Allergic reactions
8	URI	Immunizations	GID	Intestinal infection
9	Congenital anomalies	Perinatal conditions	Gastroenteritis	Headache
10	DM	Pneumonia	Hypertension	Hypertension

Epilepsy = epilepsy, convulsions; CVCA = cardiac and circulatory congenital anomalies; URD = other upper respiratory disease; Anemia = deficiency and other anemia; Jaundice = hemolytic jaundice and perinatal jaundice; SLE = systemic lupus erythematosus and connective tissue disorders; URI = other upper respiratory infections; Congenital anomalies = other congenital anomalies; DM = diabetes mellitus without complication; Immunizations = immunizations and screening for infectious disease; Perinatal conditions = other perinatal conditions; Pneumonia = pneumonia (except that caused by tuberculosis or sexually transmitted disease); Tonsillitis = acute and chronic tonsillitis; Admission = administrative/social admission; GID = other gastrointestinal disorders; Gastroenteritis = noninfectious gastroenteritis; Hypertension = essential hypertension; Headache = headache, including migraine.

**Table 4:** Top 10 most common diagnoses of adult ambulatory care by hospital level, 2008–2011.

Top 10 Order	Academic Medical Center	Metropolitan Hospital	Local Community Hospital	Physician Clinic
1	Epilepsy	Jaundice	URI	URI
2	CVCA	URI	Jaundice	Acute bronchitis
3	Jaundice	Allergic reactions	Acute bronchitis	Tonsillitis
4	Anemia	URD	Immunizations	URD
5	DM	Immunizations	Tonsillitis	Gastroenteritis
6	Congenital anomalies	Acute bronchitis	URD	Gastritis and duodenitis
7	SLE	Pneumonia	Allergic reactions	LRD
8	URI	Admission	Admission	Hypertension
9	Asthma	Tonsillitis	Pneumonia	Allergic reactions
10	URD	FUO	GID	Headache

Epilepsy = epilepsy, convulsions; CVCA = cardiac and circulatory congenital anomalies; Jaundice = hemolytic jaundice and perinatal jaundice; Anemia = deficiency and other anemia; DM= diabetes mellitus without complication; Congenital anomalies = other congenital anomalies; SLE = systemic lupus erythematosus and connective tissue disorders; URI = other upper respiratory infections; URD = other upper respiratory disease; Immunizations = immunizations and screening for infectious disease; Pneumonia = pneumonia (except that caused by tuberculosis or sexually transmitted disease); Admission = administrative/social admission; Tonsillitis = acute and chronic tonsillitis; FUO = fever of unknown origin; GID = other gastrointestinal disorders; Gastroenteritis = noninfectious gastroenteritis; LRD = other lower respiratory disease; Hypertension = essential hypertension; Headache = headache, including migraine.

## Supplementary Materials

The following are available online at http://www.mdpi.com/1660-4601/15/7/1538/s1, Figure S1: Ranking figure of the top 10 most common diagnoses of adult ambulatory care in academic medical centers, 2000–2011, Figure S2: Ranking figure of the top 10 most common diagnoses of adult ambulatory care in metropolitan hospitals, 2000–2011, Figure S3: Ranking figure of the top 10 most common diagnoses of adult ambulatory care in local community hospitals, 2000–2011, Figure S4: Ranking figure of the top 10 most common diagnoses of adult ambulatory care in physician clinics, 2000–2011, Table S1: Poisson regression analysis between variables (diagnosis and year of diagnosis) and the incidence rate ratio of adult ambulatory visits to pediatric clinics.
