# Supplementary material for: Visits to Pediatric Clinics by Adult Patients: A Nationwide Survey in Taiwan
Source: Int J Environ Res Public Health. 2018 Jul 20;15(7):1538. doi: 10.3390/ijerph15071538 (PMC6069158; doi:10.3390/ijerph15071538)
Supplement: Supplementary file 1 [file ijerph-15-01538-s001.pdf]

Supplement Figure 1. Ranking figure of top 10 most common diagnosis of adult ambulatory care in academic medical center, 2000-2011.

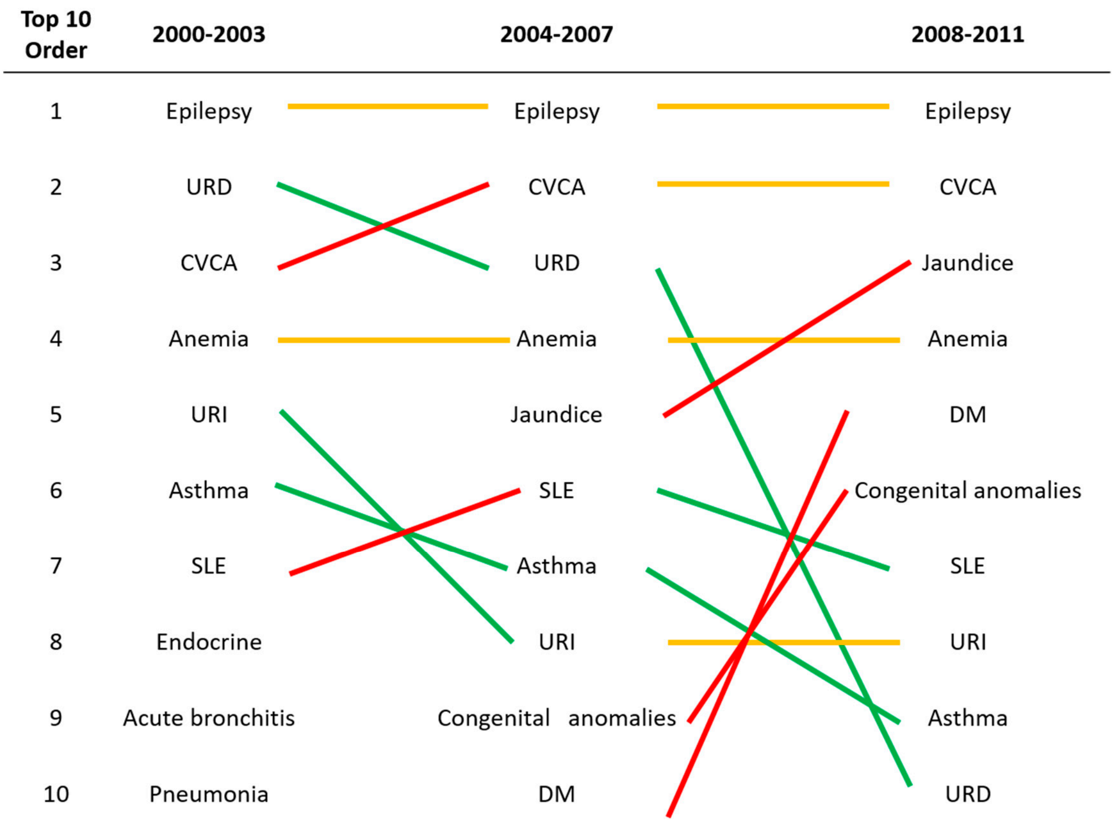

Supplement Figure 2. Ranking figure of top 10 most common diagnosis of adult ambulatory care in metropolitan hospital, 2000-2011.

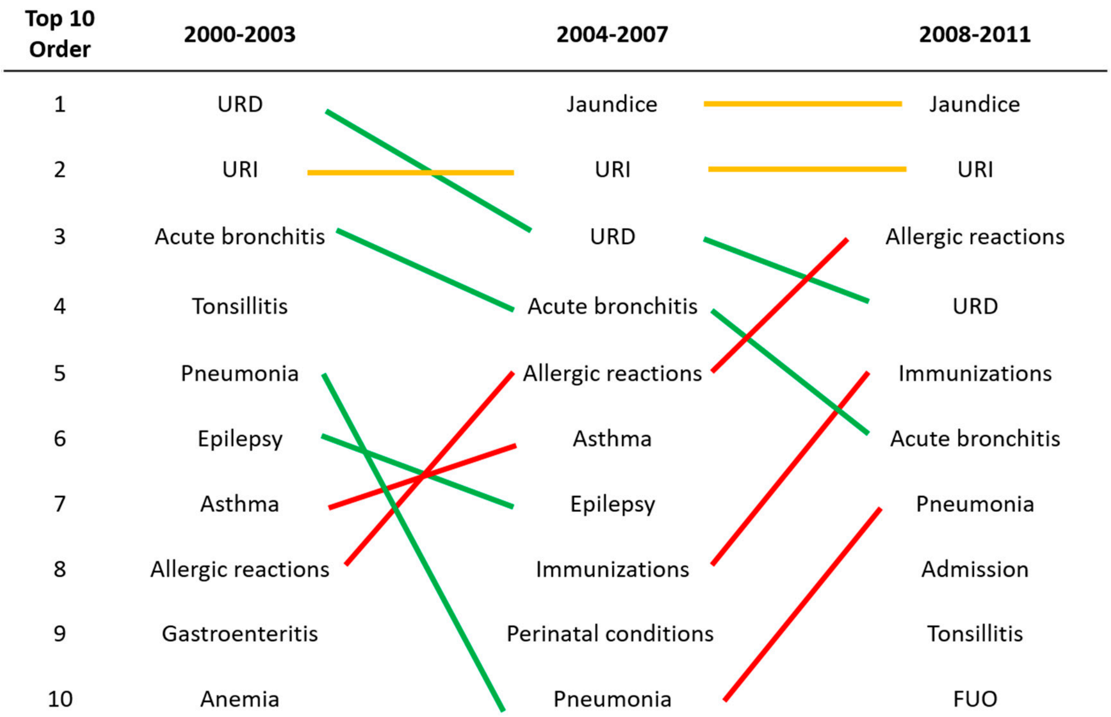

Supplement Figure 3. Ranking figure of top 10 most common diagnosis of adult ambulatory care in local community hospital, 2000-2011.

Top 10 Order	2000-2003	2004-2007	2008-2011
1	URI	URI	URI
2	URD	Jaundice	Jaundice
3	Acute bronchitis	Acute bronchitis	Acute bronchitis
4	Asthma	Tonsillitis	Immunizations
5	Allergic reactions	URD	Tonsillitis
6	Pneumonia	Admission	URD
7	Tonsillitis	Allergic reactions	Allergic reactions
8	Gastroenteritis	GID	Admission
9	Hypertension	Gastroenteritis	Pneumonia
10	CVD	Hypertension	GID

Supplement Figure 4. Ranking figure of top 10 most common diagnosis of adult ambulatory care in physician clinic, 2000-2011.

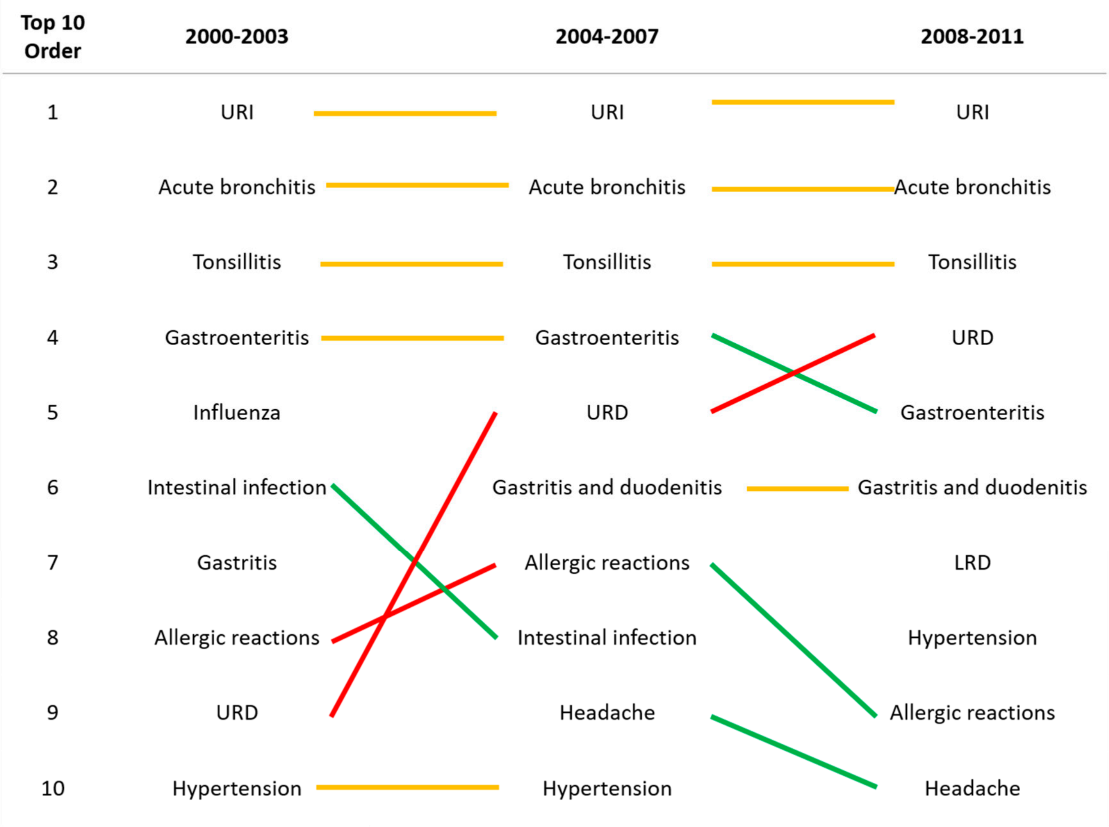

Supplement Table. Poisson regression analysis between variables (diagnosis and year of diagnosis) and the incidence rate ratio of adult ambulatory visits to pediatric clinics

Variable	IRR	95% CI	
Diagnosis			
Allergic reactions	1.00		
Acute and chronic tonsillitis	3.66	3.54-3.79	*
Acute bronchitis	5.31	5.14-5.49	*
Essential hypertension	0.74	0.71-0.78	*
Gastritis and duodenitis	2.30	2.21-2.40	*
Headache; including migraine	2.00	1.91-2.09	*
Intestinal infection	6.60	6.32-6.89	*
Noninfectious gastroenteritis	3.67	3.53-3.81	*
Other upper respiratory disease	2.89	2.79-3.00	*
Other upper respiratory infections	3.89	3.78-4.01	*
Year			
2000-2003	1.00		
2004-2007	1.48	1.45-1.50	*
2008-2011	1.99	1.96-2.02	*

IRR = incidence rate ratio; CI = confidence interval; * $p < 0.01$.
